# Making Referents Seen and Heard Across Signed and Spoken Languages: Documenting and Interpreting Cross-Modal Differences in the Use of Enactment

**DOI:** 10.3389/fpsyg.2022.784339

**Published:** 2022-07-22

**Authors:** Sébastien Vandenitte

**Affiliations:** LSFB-Lab, Department of French and Romance Languages and Literatures, NaLTT, University of Namur, Namur, Belgium

**Keywords:** enactment, sign language, gesture, depiction, multimodal, comparative linguistics, comparative semiotics

## Abstract

Differences in language use and structures between signed and spoken languages have often been attributed to so-called language “modality.” Indeed, this is derived from the conception that spoken languages resort to both the oral-aural channel of speech and the visual-kinesic channel of visible bodily action whereas signed languages only resort to the latter. This paper addresses the use of enactment, a depictive communicative strategy whereby language users imitate referents in signed and spoken languages. Reviewing comparative research on enactment, this paper highlights theoretical and methodological shortcomings in prior works. First, a broader set of causal explanations needs to be taken into account when interpreting differences between signing and speaking communities. A more comprehensive conceptual toolbox ensures that differences are not automatically attributed to modality. In particular, less-studied factors of language diversity, like sociolinguistic and cultural ecologies, and how they interact with other factors should be considered. Second, diversity in enactment across signed and spoken languages is shown to be inadequately and insufficiently documented. It is argued that by comparing enactment across more diverse signing and speaking communities and using large, directly comparable corpora, solid analyses can be carried out, enabling a better understanding of how and why different communities use enactment in similar or different ways.

## Introduction

Enactment is a communicative strategy used in many languaging communities.^1^ When enacting, a language user denotes a referent and the latter’s actions (bodily behaviour, emotions, thoughts, utterances) using depiction, a method of signaling exploiting perceptual resemblances between communicative forms and their meanings (Clark, 1996; Cormier et al., 2015). This is done by means of bodily movements like the use of gaze, facial expression, torso and hand movements as well as voice (Clark and Gerrig, 1990). The phenomenon has received other labels such as “character viewpoint gesture,” “role shift” and “constructed action,” among others (McNeill, 1992; Herrmann and Steinbach, 2012; Cormier et al., 2015). This terminological multiplicity is paired with a lack of consensus as to how enactment can best be described by linguists. One reason for this lies in the differences in how enactment is used in signed and spoken languages (hereafter, “SLs” and “SpLs”).

In the present paper, I start by introducing the framework adopted here to compare SLs and SpLs, i.e., a comparative semiotics of signers’ and speakers’ signalling repertoires (Kendon, 2014; Ferrara and Hodge, 2018). The paper then focuses on the comparative semiotics of enactment and describes studies that address and compare the phenomenon across SLs and SpLs. Next, I review these works and lay out a research agenda for enactment articulated around Cooperrider’s (2019, p. 211) proposal for a double shift in the study of gesture diversity and universals:

*First, we need better data. We can’t afford to overgeneralize on the basis of thin, scattershot descriptions. We need data from culturally and geographically disparate communities, and we need such data to be systematically collected for comparative purposes. And yet data on its own is not enough. The second thing we need is better, more explicit conceptual frameworks. That is, we need intellectual tools to help us make sense of the data we already have, as well as to guide the collection of more data.*

Next, the paper addresses the main approaches taken to account for cross-modal differences. Based on a review of the literature and drawing from like-minded works, I argue that causal accounts of cross-modal differences have been too narrow in scope. I stress the interplay of multiple differences between signing and speaking communities that should be considered when explaining communicative diversity. In addition, I show that comparative research on enactment has been based on small data samples of sometimes monologic, non-spontaneous language use. After arguing that these issues undermine our understanding of enactment across SLs and SpLs, I expand on one way to test claims about the factors which cause cross-modal differences: the use of directly comparable corpora of SLs and SpLs (Johnston, 2013; Hodge et al., 2019). Finally, a summary of the points made in the paper is provided.

### Comparing Signed and Spoken Languages

How can signers’ and speakers’ languaging practices be compared? First, SLs should be compared with speech-gesture ensembles rather than speech only (Vermeerbergen and Demey, 2007). A second step is that of operationalising the comparison on a modality-free basis (Okrent, 2002). Communicative moves in both categories of languages are composites involving different methods of communication that correspond to the uses of Peirce’s (1955) three types of signs: symbols, indices and icons (Clark, 1996; Enfield, 2009; Ferrara and Hodge, 2018). Description consists in the use of symbols, i.e., often arbitrary, conventionalised form-meaning pairs, such as lexical and morpho-syntactic constructions or emblematic gestures (Clark, 1996; Teßendorf, 2013). Indicating a referent comes down to anchoring it to a time and place (Clark, 1996). Pointing constitutes one type of indication whereby language users provide an instruction to their audience to look for the referent physically anchored by the extended finger or body part (Clark, 2003). Finally, depiction consists in exploiting the perceptual resemblance between a form and what it denotes to give one’s addressee a near first-hand experience of a referent (Clark, 1996, 2016). Depiction is well-studied in speakers’ gestures (e.g., McNeill, 1992) but is increasingly recognised as crucial in speech and SLs too (Ferrara and Hodge, 2018). For instance, in languages like Siwu or Japanese, ideophones are lexical classes made up of words which “*depict sensory imagery*” (Dingemanse, 2019, p. 21). Hence, both SLs and SpLs are actioned by means of description, indication and depiction, three methods of communication which rely on different processes. Within a comparative semiotic approach, one can ask: How is the use of these methods distributed across different speaking and signing communities? How are they combined? In what contexts? (Ferrara and Hodge, 2018). Our focus now turns to a comparative semiotics of enactment.

### Comparing Enactment in Signed and Spoken Languages

**FIGURE 1:**
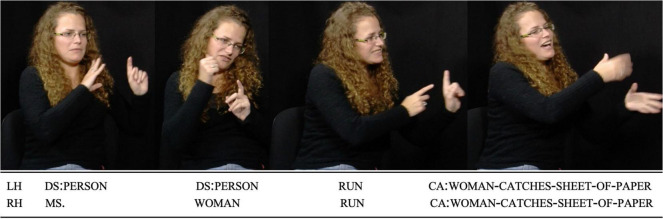
LSFB Corpus, Session 29, S059, Task 12: 00:04:00.002 – 00:04:03.895. Reproduced with permission. The woman starts running and catches the sheet of paper, relieved.

**FIGURE 2:**
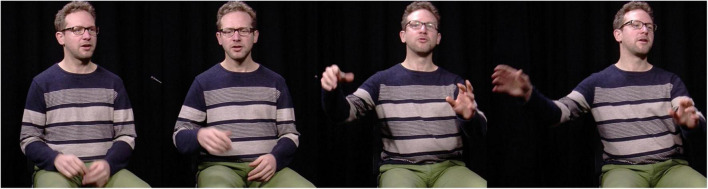
FRAPé Corpus, Session 10, L019, Task 12: 00:00:20.074 – 00:00:21.681. Reproduced with permission. [Il y a une jeune dame aussi qui a l’air de partir au travail] qui qui court après un papier. [There’s also a young woman who seems to be leaving for work] who who’s running after a sheet of paper.

Figures 1, 2 exemplify the use of enactment in LSFB (*Langue des Signes de Belgique francophone*, French Belgian Sign Language)—the SL of the deaf community who lives in the Brussels and Wallonia regions of Belgium—and its ambient SpL—Belgian French. Enactment is exemplified in four utterances drawn from the LSFB and FRAPé corpora (Meurant, 2015).^2^

In Figures 1, 2, both the LSFB signer and the Belgian French speaker take part in a narrative retelling task after watching a cartoon film. Both examples zoom in on a section of the retellings where they describe a woman running to catch a sheet of paper blown away by the wind. Both language users rely on description to talk about the woman (“*MS,”* “*WOMAN”* in LSFB and “*woman*” for French in a clause preceding the one featured in the example) and the running event (“*RUN”* in LSFB and “*running*” in French). Simultaneously, they use their bodies to enact the story character’s actions. While uttering the lexical sign “*RUN”*, the LSFB signer draws the woman’s worried facial expression and rotates their head and torso to the left. The signer’s gaze is shifted in the same direction, away from the addressee (see third still). Finally, the signer extends their arms to imitate the catching and draws a relieved facial expression (fourth still). In the Belgian French example, while uttering “*who who’s running after a sheet of paper*,” the speaker simultaneously enacts the woman using several articulators: they rotate their head to the right, redirect their gaze upwards and move their arms to enact how the woman tries to catch the sheet of paper (second, third and fourth stills). These examples show that signers and speakers can use enactment to depict the same event (see also Johnston, 2013, p. 118, for an example of similar uses of enactment by an Auslan [Australian Sign Language] signer and a speaker in McNeill, 1992).

**FIGURE 3:**
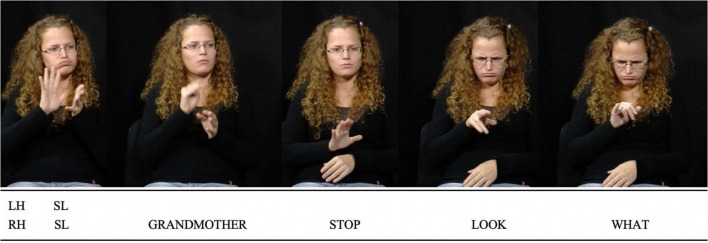
LSFB Corpus, Session 29, S059, Task 5: 00:03:47.596 – 00:03:50.587. Reproduced with permission. We were signing and my grandmother waved at me: “Stop (signing)”. I looked at her and was like: “What?”.

In Figure 3, the signer retells an interaction with their grandmother on a bus. After signing for a while, the grandmother experiences linguistic insecurity due to marginalising attitudes towards SLs (Padden and Humphries, 2006; Hill, 2015). The signer is then told by their grandmother to stop signing. In the example, the signer enacts the grandmother by reorienting their head and breaking gaze address while producing the sign glossed “STOP” (third still). In the remainder of the illustration (fourth and fifth stills, co-occurring with “*LOOK*” and “*WHAT*”), the speaker enacts their own reaction by adopting an incredulous facial expression, leaning their head forwards and quoting themselves: “*WHAT?*”

**FIGURE 4:**
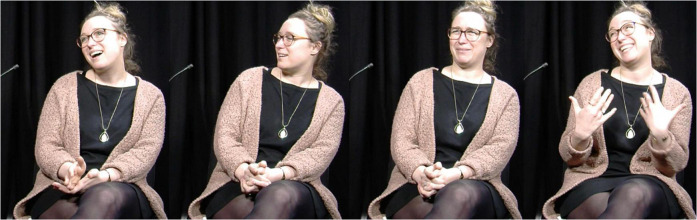
FRAPé Corpus, Session 10, L020, Task 5: 00:04:27.620 – 00:04:35.071. Reproduced with permission. “euh il drache euh c’est”, donc là je vois Guillaume qui tique: (inintelligible) “il drache ?” “Ok Guillaume en fait, (ça veut dire) ‘il pleut très très fort’ en Belgique”. “erm it’s lashing down erm it’s”, so then I see Guillaume flinch: (unintelligible) “it’s lashing down?”. “Ok Guillaume so, (it means) ‘it’s raining lots and lots’ in Belgium”.

In Figure 4, the speaker discusses lexical variation between Belgian and Canadian French and retells an event where, upon their using the Belgian French word *drache* (“rain”), a Canadian colleague is left wondering what they mean. The speaker first enacts themselves announcing that it is raining (*“it’s lashing down”*) (first still). Next, this enactment continues as the signer utters “*so then I see Guillaume*”: they turn their head to the right side, lean their upper body towards the left side and redirect their gaze as though looking at their colleague (second still). They then use enactment while uttering Guillaume’s words “*it’s lashing down?*” to show their colleague’s puzzled facial expression (third still). Finally, they enact themselves responding to the colleague, changing their gaze direction, and adopting a smiling facial expression while explaining the meaning of *drache* (fourth still): “*Ok Guillaume so, (it means) ‘it’s raining lots and lots*’.”

In the present paper, “enactment” is used as an umbrella term to refer to all uses of this strategy by both signers and speakers. This is motivated by the assumption that a same phenomenon underlies at least some instances of enactment in both SLs and SpLs and that “enactment” can be used as a label for a comparative concept (Haspelmath, 2010; Hodge and Cormier, 2019). Research has revealed that enactment is part and parcel of the repertoires of both signing and speaking communities (McNeill, 1992; Quinto-Pozos, 2007; Ferrara and Johnston, 2014; Thompson and Suzuki, 2014; Clark, 2016). Linguists and gesture researchers have tried to explore how enactment varies across semantic domains or referential targets, discourse genres, and other factors of variability in language use. Much attention has been given to quotational phenomena. Indeed, enactment often co-occurs with discourse chunks in which language users refer to utterances and, in SLs, it may be the most standard way to refer to a referent’s utterances (Quer, 2011; Herrmann and Steinbach, 2012; Hodge and Cormier, 2019). In SpLs, enactment is very frequent in speech reports too (Clark and Gerrig, 1990).^3^ Though several have argued that utterance reporting constitutes a sub-kind of enactment, inasmuch as the reported utterance is an action that is depicted (Clark and Gerrig, 1990; De Brabanter, 2017), these two notions are distinguished to easily expose diverging perspectives in the literature. Hence, like Steinbach (2021), I here distinguish between “quotational” enactment, the use of visible bodily articulators and voice to depict a referent whose utterances are reported, and “non-quotational” enactment, the use of the same channels to depict a referent performing actions different from languaging. In Figures 3, 4, two instances of utterance reporting are found in LSFB (“*STOP,”* “*WHAT”*) and three in French (“*it’s lashing down*,” “*it’s lashing down?*” and “*Ok Guillaume so, [it means] ‘it’s raining lots and lots’*”). These utterance reports all co-occur with quotational enactment as language users depict the referents whose utterances are reported using their gaze, head, torso, facial expression and voice. The two examples also feature non-quotational enactment where the referents’ actions are depicted, but not their utterances. These tokens of non-quotational enactment co-occur with the production of “*LOOK”* in LSFB and “*I see Guillaume”* in Belgian French.

While both quotational and non-quotational uses involve the perspective-taking of another referent or of the language user in another context (e.g., time or place), researchers have addressed or integrated these phenomena in different ways. On the one hand, some have approached enactment based on Clark and Gerrig’s (1990) account of quotation as depiction (e.g., Hodge and Cormier, 2019). This approach sees the “reporting” or “construction” of utterances or thoughts and of other actions as forms of depiction. Following this account, it is no surprise that these uses present many similarities in SpLs and SLs (e.g., Liddell and Metzger, 1998). On the other hand, other researchers have taken a particular interest in providing formal accounts of the phenomenon. By these accounts, some properties of enactment in SLs cannot be fully explained by an approach treating the phenomenon only as quotation or depiction (Lillo-Martin, 2012). For instance, looking at the behaviour of indexical expressions, researchers have claimed that indexical shifts occur beyond quotational contexts or that some place and time indexicals do not always get a shifted interpretation in enactment (e.g., Quer, 2011). Therefore, they argue, at least some uses of enactment in SLs could rather be likened to (language-specific) conventionalised structures in SpLs.

Moving to the varied discourse genres in which enactment is found, the phenomenon has been widely described for narrations in both SLs and SpLs (Hodge and Ferrara, 2014; Stec et al., 2016; Bressem et al., 2018). One function of enactment in the narrative genre has to do with the liveliness enabled by this referential strategy (Tannen, 2007). Since enactment provides addressees with a seeming first-hand experience of a referent and/or the action(s) performed by the latter, it is particularly useful for what Clark and Gerrig (1990, pp. 793–794) call “engrossment”: “*Direct and indirect quotation contrast in whose perspective the addressees are to get engrossed in.* […] *On the addressee’s side, to become engrossed in an event is to reexperience it vividly.*” Clark and Gerrig’s observations are echoed in research carried out on how enactment can be used as a resource for viewpoint expression and relies on embodied simulation (e.g., Dancygier and Vandelanotte, 2017; Hostetter and Alibali, 2008). For instance, enactment can be used by interactants to display affective or evaluative stance, e.g., distancing themselves, with respect to the enacted referent and/or behaviour (e.g., Niemelä, 2010; Shaffer, 2012; Palfreyman, 2020).

A substantial part of the literature has documented the use of enactment in narration and/or for the expression of stance. It is however clear that enactment is also a useful way to represent referents (Slonimska et al., 2021). As Clark and Gerrig (1990, p. 793) point out, enacting a referent is a good solution to the issue of ineffability (see also Quinto-Pozos, 2007 for a discussion of how enactment can be considered as obligatory for similar reasons in ASL):

*Many things are easier to demonstrate than describe. Imagine trying to describe how to tie a shoe, parry a lunge in fencing, or knit purl.* […] *It is also generally easier to demonstrate: emotion, urgency, indecision, and sarcasm in tone of voice; gestures, facial expressions, or other body actions;* […]. *If speakers and addressees try to minimize effort in communication, as generally assumed* […], *whether speakers describe or demonstrate an aspect should depend, all else being equal, on which is easier*.

Even though enactment is more frequent in narration than in conversational settings (Puupponen et al., 2022), it is by no means confined to this genre. A growing body of research has shown how enactment can prove a powerful tool to do reference in SLs. In that respect, one aspect that has drawn signed language linguists’ attention is the extent to which enactment co-occurs with other semiotics. For instance, Cormier et al. (2015, p. 189) propose a three-way typology of the phenomenon distinguishing between “overt,” “reduced” and “subtle” enactment (see also Quinto-Pozos and Mehta, 2010): overt enactment exhibits “*strong intensity*,” the use of “*many articulators*” and does not co-occur with other elements of “*narration.*” When producing overt enactment, language users fully adopt the enacted referent’s perspective. When language users produce reduced enactment, by contrast, they resort to few articulators and use “*simultaneous narration (lexical material).*” In such cases, language users’ alignment with the perspective of the enacted referent is weak. Subtle enactment lies in between these two categories. While language users are mostly aligning with the enacted referent’s viewpoint, their own internal perspective, though less salient, remains active due to the production of “*some simultaneous narration (lexical material)*.” While this division has been revised (Jantunen et al., 2020), the recognition of different degrees of enactment has proved informative to document how the phenomenon varies across registers. Indeed, Puupponen et al. (2022) show that, though overt enactment is the most frequent type in both FinSL (Finnish Sign Language) conversational and storytelling settings, the distribution of overt and non-overt enactment varies as a function of discourse genre.

Looking at “clause-like units” in Auslan, Ferrara and Johnston (2014) and Hodge and Johnston (2014) have shown that enactment can be the only strategy expressing core information like the denoted process, the participant (be it agent or patient) involved in the said process, or even the combination of these two pieces of information (see also Jantunen, 2017 on the interplay of enactment and FinSL clauses). Whereas it is clear that less conventional semiotics merge with spoken discourse, making “composite utterances” emerge (Enfield, 2009), the interaction of enactment with SpL structure has received less attention. Some studies have pointed out that enactment can fit within the organisation of clauses in SpLs. For instance, as Clark and Gerrig (1990) show in “*The boy who had scratched her Rolls Royce went [rude gesture with hand] and ran away*.” (p. 781), tokens of enactment can function as constituents embedded in SpL utterances (see also De Brabanter, 2010; Ladewig, 2020). Enactment is notably useful when the utterer’s intended information is particularly dense (Slonimska et al., 2021). Seeking to single out referential functions of LIS (Italian Sign Language) enactment, Slonimska et al. (2021) use a controlled experimental setting where participants play a director-matcher game in pairs. After one player is asked to describe images varying in the amount of information (ranging from two to five information units) they display, the other player is asked to retrieve the corresponding images based on their description. Slonimska et al. (2021) note that increases in the amount of information found in the images lead to a higher use of enactment, hence suggesting that enactment is used by LIS signers for communicative efficiency. One explanation for this lies in signers’ simultaneous use of different bodily articulators, notably for enactment (Quinto-Pozos and Parrill, 2015; Slonimska et al., 2020). As Slonimska et al. (2021, p. 372) exemplify,

*A signer who tilts their head upwards while depicting a person shaking hands does not only intensify the depiction of the character but also provides information in its own right, i.e., that the character is shorter than the person [they are] shaking hands with.*

The example provided by Slonimska et al. (2020) puts in the foreground another, related, property of enactment: when enacting referents, language users can also convey information about other referents. Indeed, gaze direction, body posture and other bodily behaviours can index another referent to be construed for addressees. Liddell (2003) coins these referents “invisible surrogates.” Using an example from ASL (American Sign Language) where a signer reports an interaction between two referents, Liddell exemplifies how the utterance “*KNOW WHERE MY HOME*” (translated as “*Do you know where my home is?*”) is produced with cues which indexically refer to the addressee being asked the question: “*the signer does not merely recite the signs of the questioner. He directs his eye gaze towards an imagined addressee*” (2003, p. 159). Padden (1986, p. 50) refers to these instances of enactment where two referents are construed as “contrastive role shifting.” Herrmann and Steinbach (2012, p. 220) propose that, when DGS (German Sign Language) signers report conversations between two interactants, they position several bodily articulators with respect to locations in the signing space associated with the reported utterer and addressee:

*The loci of the signer and the addressee of the reported utterance act as anchors for the respective perspective shifts during quotation.* […] *[T]he signers’ eye gaze and the head are turned towards the addressee of the quoted situation* […]. *By contrast, the signers’ body leans towards the locus of the quoted signer.*^4^

This argumentation resorts to an important body of literature on SLs’ spatial reference-tracking systems. For instance, Lillo-Martin and Klima (1990) describe how ASL signers associate specific referents with positions in the space surrounding them, using so-called “referential loci,” and subsequently exploiting them in discourse. Puupponen (2019, p. 16) also notes that movements of the head or body can indicate referents, notably in tokens of enactment:

*In some movements of the head or the whole upper body, the head moves towards an introduced or previously established location (i.e., an imaginary referent) while the face and gaze may be oriented towards an addressee* […] *or to the imaginary referent*. […] *[T]hese movements anchor the simultaneously occurring manually signed contents to the reference point of an imaginary referent*.

Similar indexical properties of enactment are certainly present in SpLs. Comparing the first and the second still of Figure 4, it is striking that the speaker shifts from a neutral position to the use of the same bodily articulators (gaze, head, and upper body) to enact themselves looking at their Canadian colleague, thereby indexing the latter’s position in the depicted scene.

Summing up, a multifaceted picture of the contexts of use, semiotic functions and forms of enactment across SLs and SpLs has been presented. Despite striking similarities, such as the use of the same bodily articulators and its frequent occurrence in narration and quotational contexts, the comparison of enactment across SLs and SpLs remains a topic of debate. It remains unclear to what extent signers’ and speakers’ uses of enactment constitute the same phenomenon. These claims are spelled out in more detail in the next section.

## Cross-Modal Differences in Enactment

### Frequency of Use

Researchers have described enactment as highly frequent in SLs, more so than in SpLs. Herrmann and Pendzich (2018, p. 285) claim that one cannot “*find the identical frequency of such comparable strategies in spoken language.*” In a similar vein, the use of enactment in utterance reports has been described as obligatory inasmuch as enactment “*necessarily marks the linguistic report*” (Quer, 2019, p. 225). Despite claims of pervasiveness in SLs, few studies quantify the occurrence of enactment. Hodge and Ferrara (2014)’s study of Auslan narrative text-based and picture-based retellings is an exception. They note that roughly 39% of the discourse time co-occurs with enactment. It is less clear how frequent the strategy is in SpLs. In their study on American English, Stec et al. (2016) find that nearly all 704 (97.4%) tokens of utterance reporting in their dataset of personal narratives co-occurred with enactment.

Studies comparing SLs with their ambient SpLs attest that enactment is more frequent in the former. Rayman (1999) compares ASL and English narrative retellings and reports that ASL signers “*reliably* [used] *role-shifting throughout the story* [while] *English speakers did not enter into the role of either of the characters.*” Marentette et al. (2004) compared narrative retellings by ASL signers and English speakers and also found that productions in ASL exhibited a more frequent use of enactment than the ones in English. Focusing on BSL (British Sign Language) and British English storytellers, Earis and Cormier (2013, p. 340) found that “*depicting characters using expressive elements such as co-speech gesture does not always occur in spoken English, but depicting characters* […] *appears to be a very important element of storytelling in signed narratives.*” Finally, Quinto-Pozos and Parrill (2015) compared how ASL signers and English speakers used depiction relying on either internal viewpoint—enactment—or external viewpoint. Their depictions were elicited by exposing participants to specific events of the same narrative in a retelling task of short cartoon clips. Those events which led speakers to use enactment were also retold by signers using the same strategy. Nevertheless, for those events that speakers depicted from an external viewpoint, signers not only used external viewpoint but also produced enactment. One may thus infer that ASL signers used it more than English speakers overall. Hence, based on those language pairs that have been compared, enactment is more frequent in SLs than in SpLs.

### Use of Enacting Articulators and Manners of Articulation

Another cross-modal difference lies in the articulators used for enactment. The most frequently mentioned articulators for SLs are gaze, face, head and torso, often subsumed as “non-manuals.” In Herrmann and Steinbach (2012)’s study on DGS utterance reporting, they report the following frequencies for these articulators: face (98%), gaze (86%), head (77%) and body lean (48%). For ASL, Quinto-Pozos and Parrill (2015) code for three categories: “affect” (namely, the use of facial expressions), “torso” and “handling” (i.e., the use of a language user’s hand(s) to depict the manipulation of an object). Quinto-Pozos and Parrill report a total of 176 enactment tokens. In 150 (85.2%), face was used whereas torso and signers’ hands were used in respectively 113 (64.2%) and 61 (34.7%). While the use of handling is restricted to those events which equally elicit enactment in speakers’ renditions, the use of facial expression and torso movements are prevalent in all kinds of events, leading to the conclusion that “*uses of affect and the torso by signers are common and important ways to engage in the retelling or narration of a set of events*” (Quinto-Pozos and Parrill, 2015, p. 27). It is clear that non-manuals play an important role in signed enactment. But this may not be a modality-specific result. In Stec et al. (2016)’s study of American English quotational enactment, non-manuals were found to be more frequent than the enacting use of hands. Posture change (arguably involving both head and torso movements), gaze and face were used in, respectively, 84.7, 71.4, and 47.7% of tokens. In comparison, hands were only used in 20.6% of the tokens. It is also worth noting that Stec et al.’s study addressed a SpL and hence included the analysis of voice, which was used in 55.3% of tokens.

Still, differences across SLs and SpLs are corroborated by studies making cross-modal comparative claims. Rayman (1999, p. 78) notes that “*in contrast* [to deaf ASL signers], *the hearing narrators rarely used facial expression to depict characters.”* Non-manuals, including gaze as well as head and torso, are also cited as more frequent in BSL than British English enactment (Earis and Cormier, 2013). According to Herrmann and Pendzich (2018), in DGS non-quotational enactment too, there are cross-modal differences in the recruited articulators: unlike DGS signers, speakers use their legs to enact referents. Further work may be warranted as the use of the lower half of the body has been attested in other SLs, like ASL, FinSL or LSFB (Quinto-Pozos and Mehta, 2010; Jantunen et al., 2020; Vandenitte, 2021). Hence, though it is not clear which specific articulators are more frequent in SLs and SpLs, prior research suggests that signers resort to specific articulators more systematically than speakers do.

In addition to the use of specific articulators or absence thereof, there have been claims that even when signers and speakers resort to similar articulators, the latter may still be used in different ways. Hence, SLs and SpLs may also differ in the manners of articulation recruited for enactment. Specifically, some uses of enactment, like quotational uses, in SLs have been described as exhibiting more systematic formal characteristics. Quer (2019, p. 225) proposes that enactment “*occurs systematically intertwined with the utterances or thoughts of the reported agent, in a richer and more structured fashion than in co-speech gesture.*” The systematic use of role-shift, whereby language users map referents in the space around them and use that space in enactment, has also been argued to be specific to SLs. Padden (1986, p. 49) says:

*“Role shift” is perhaps an unfortunate term*. […] *These kinds of descriptions incorrectly suggest that whatever common sense knowledge we have about play-acting ought to apply to understanding how role-shifting works in ASL.*

In several works comparing SLs and SpLs, differences related to role-shift patterns are mentioned. In Earis and Cormier (2013), the use of eye gaze as well as head and torso positioning is reported to be more conventionalised in BSL than British English. Whereas speakers’ use of enactment is largely seen as depictive, BSL signers orient their eye gaze, head and torso in order to align with locations in the signing space associated with specific referents. Perniss and Özyürek (2015) compare the use of enactment predicates, i.e., iconic lexical signs denoting manual actions, in DGS with enacting hand movements performed by German speakers when retelling addressees about stimuli consisting in video vignettes. In the same line as Earis and Cormier, Perniss and Özyürek (2015, p. 51) note that

*DGS signers perform Enactment predicates depicting manual manipulation (e.g., unscrewing the lid of a jar) at the location associated with the referent performing that action nearly half the time* […]. *In contrast, German co-speech gesturers very rarely localize these types of Enactment predicates*.

To sum up, signers and speakers seem to use enactment in different ways. Signers use enactment more frequently. In addition, signers might exhibit more conventionalised enactment practices inasmuch as they may more consistently use certain articulators, locations and/or modes of articulation. In the remainder of this paper, I will come back to these studies, review their methodologies and argue that further work is needed to ascertain that claims on enactment conform with the ways language users enact referents in naturally occurring discourse. Before that, some ways in which these potential differences have been and could be accounted for are laid out.

## Accounting for Cross-Modal Differences

### From Modality Effects to a Broader Set of Causal Frames

What is to be made of differences between signers and speakers? How can they be accounted for? Enfield (2014, p. 13) provides a framework to account for linguistic diversity based on six causal frames:

*Each of the six frames – microgenetic, ontogenetic, phylogenetic, enchronic, diachronic, synchronic – is distinct from the other in terms of the kinds of causality it implies, and thus in its relevance to what we are asking about language and its relation to culture and other aspects of human diversity. One way to think about these distinct frames is that they are different sources of evidence for explaining the things that we want to understand.*

The following sections present the frames deemed most relevant to account for cross-modal differences. I exemplify how these frames have been used to account for cross-linguistic and cross-modal differences broadly (first addressing languaging phenomena that are separate from enactment). Next, the discussion narrows down to how Enfield’s framework can help reframe the interpretation of cross-modal differences in enactment.

#### The Microgenetic Frame

Microgeny refers to the phenomena usually studied in the fields of psycho- and neurolinguistics, phonetics and kinematics. Enfield subsumes it as the set of processes linked to the ways in which humans process actions. Because speech communities process languages in very similar ways, accounts of differences based on microgenetic causes are rare. Still, Moisik and Dediu (2017, p. 1) use a biomechanical model to examine “*whether variation in human vocal tract anatomy and physiology constitutes a systematic bias or pressure on speech sound systems.*” In particular, this hypothesis is examined by investigating the link between the presence of click sounds in the phonological system of a language and aspects of alveolar ridge morphology. Microgenetic causal explanations have also been used to explain diversity in gesture. For instance, Cooperrider et al. (2018) observe that non-manual pointing is distinctively prevalent in the Yupno speaking community. Some causes offered to account for this prominence are the pressure towards minimal effort or potential long-term effects of speakers’ hands being frequently occupied (e.g., performing manual work) during interactions, hindering the use of manual pointing.

The differences between signing and speaking communities are sometimes depicted as owed to so-called “modality” effects. In this tradition, microgenetic factors are invoked: SLs are often described as visually perceived and kinesically produced whereas SpLs additionally rely on vocal production and auditory perception (Meier, 2002). Modality effects are understood as resulting from the fact that “*the language modality—auditory-vocal or visual-gestural—influences linguistic structures in different ways*” (Zeshan and Palfreyman, 2020, p. 531). One clear example of how this microgenetic difference impacts SpL and SL use has to do with their main channels: the motor controls required for the different modalities (e.g., the effort required to move one’s hands with respect to one’s vocal folds) lead to different articulatory rates (Bellugi and Fischer, 1972).

Another example has to do with the fact that interactions in signing communities take place in contexts that are somewhat different with respect to those of speaking communities. Referring to this as “*the semiotic umwelt of signers*,” Johnston (1996, p. 63) says that “*sign languages are face-to-face languages rooted in the immediate physical situation of the context of utterance to an extent seldom appreciated by non-signers. When signing, one must always be in view of one’s interlocutor and stop most non-linguistic behavior.*” Many have asked to what extent the pervasiveness of face-to-face interaction and mutual visual attention for signers may have (dis)favoured the emergence and use of specific symbolic forms with respect to SpLs. For instance, the pervasive reliance on the visual-kinesic modality in signed interaction has an impact on the presence of iconicity in SLs. Johnston (1996, p. 65) continues:

*The world is primarily temporal, visual, and spatial rather than auditory*. […] *[T]he fact that our experience, as a whole, is visual, temporal, and spatial means that a language that has visual and spatial resources for representation has greater means for mapping onto itself those very visual and spatial qualities.*

Languages are, in part, shaped by the sensory and linguistic experiences of their users (but see Kusters et al., 2017 on how individuals flexibly navigate different interactional contexts using diverse semiotic resources). Interactions within communities lead to the emergence and use of forms that are tailored to their environment and communicative purposes. Though microgeny is an important frame to explain cross-modal differences, it is not the only way SpLs and SLs differ.

#### The Ontogenetic Frame

Ontogeny is the causal frame in which one looks “*at how a person’s linguistic habits and abilities are learned and developed during the course of that person’s lifetime*” (Enfield, 2014, p. 14). Ontogenetic explanations have been provided for some cross-linguistic differences. For instance, Trudgill (2009) proposes that morphosyntactic complexity is influenced by social aspects of languaging communities. Of interest here is the impact of ontogeny on what Trudgill refers to as the processes of ‘morphological complexification’ in a given language, identified by the following criteria: higher degree of “*irregularity*, *allomorphy* [as well as] *redundancy*,” as evidenced in this latter case by “*a growth in the number of morphological categories* […] *and the introduction of repetition of information”* (Trudgill, 2009, pp. 105–108). These processes have been shown to be less prominent in languages characterised by high-contact situations involving late learners of the community’s language, i.e., learners who have come in contact with this language after they had “*passed the critical threshold for language acquisition”* (Trudgill, 2009, p. 99). As Trudgill explains, morphological complexity makes it harder for late learners to master a language. Pidginisation then occurs as late language learners integrate into a community, leading to less morphological complexity. Trudgill provides several examples of morphological complexity drawn from traditional dialects of English which contrast with varieties of General English, i.e., those varieties of English involved in many high-contact situations and which count many late learners.

Schembri et al. (2018) propose that signing communities provide a good test case for the preceding ontogeny-related claims. In signing communities like the Auslan, BSL or ASL communities, deaf children are often surrounded by hearing caregivers: Mitchell and Karchmer (2004) report that this applies to more than 95% of deaf individuals. This means that there are very few native signers and that this group is in a community-internal high-contact situation with deaf non-native signers (Schembri et al., 2018). In addition, some of these non-native signers have acquired a SL as a delayed first language. Indeed, as Emmorey (2002, p. 205) states: “*[t]he critical period hypothesis has special import for the Deaf population because if a deaf infant is born to hearing parents who do not sign, then exposure to an accessible natural language will be delayed.*” This unique sociolinguistic situation makes signing communities heterogeneous groups with important internal variation, including in age of acquisition. Schembri et al. (2018, p. 5) note that following Trudgill’s criteria, SLs like Auslan, BSL and ASL can be described as having “*low to moderate levels of morphological complexity.*” This could be motivated, at least partly, by the ontogenetic factor, i.e., the “*unique sociolinguistic situation and language transmission patterns of sign languages”* (Schembri et al., 2018, p. 7). Another frame which has been shown to generate cross-linguistic differences is the enchronic frame.

#### The Enchronic Frame

The enchronic causal frame is the one in which one resorts to social-interactional processes to provide causal accounts of language phenomena (Enfield, 2014). Enchrony involves processes such as “*relevance* […], *local motives* […], *sign- interpretant relations* […], *and social accountability”* (Enfield, 2014, p. 15). For instance, in interaction, language users can enchronically choose to produce utterances depending on what has been said before, on their intentions, and on the modalities and method(s) of communication they decide to use. In making these choices, language users can be held accountable for respecting or deviating from social-interactional norms in vigour in their community. For example, research in gestural pragmatics has shown how diverse multimodal practices can be recruited for interaction. Kita (2009, p. 162) points out that “*cultures may differ in how much gesture is highlighted/foregrounded as a medium of communication.”* For instance, Japanese speakers nod more frequently than American English speakers do in conversation (Kita and Ide, 2007). This diversity has been explained enchronically by foregrounding speakers’ observance of distinct cultural norms such as differences in the “*emphasis on cooperation and consideration for others”* (Kita and Ide, 2007, p. 159). The enchronic causal frame has also been invoked to account for differences between signing and speaking communities. For instance, in narrative retelling tasks, Rayman (1999) and Marentette et al. (2004) report that signers take up more time than speakers, producing lengthier and more detailed narrations. This has been attributed to storytelling norms specific to signing communities. This is congruent with Ladd’s (2003) claim that storytelling plays a peculiarly prominent role in deaf communities like the ASL one and is considered a prestigious skill, whereby good storytellers are often seen as leaders by their peers.

#### The Diachronic Frame

Diachrony is the causal frame in which phenomena are explained in terms of *“social/cultural history* [by looking] *at elements of language as historically conventionalized patterns of knowledge and/or behaviour*” (Enfield, 2014, p. 15). Cross-linguistic lexical-grammatical distinctions have been explained resorting to diachronic processes which happen at timescales ranging from years to centuries. For instance, the language-specific processes of lexicalisation and grammaticalisation are used to account for the diversity of constructions in the world’s languages (Croft, 2001; Traugott and Trousdale, 2013). Morphological complexification is often described as a process which takes time, potentially accounting for why younger languages are less complex than older ones. This explanation has been used to account for reports that creole languages exhibit less grammatical complexity than older languages (e.g., McWhorter, 2001 but see DeGraff, 2001 for arguments against this view).

SLs are subject to the processes which feed phenomena like lexicalisation and grammaticalisation, just like SpLs (Wilcox and Occhino, 2016). For instance, the ASL lexical sign “STRONG” has undergone a grammaticalisation process whereby the sign is nowadays used as the modal “CAN” (Shaffer and Janzen, 2016). However, SLs are relatively young languages (e.g., Kyle and Woll, 1985), some still being referred to as “emerging” (Adone, 2012). Differences in language age have been provided as an explanation for differences between SLs and SpLs (e.g., Meier, 2002). The impact of SLs’ young age is however a debated issue. Aronoff et al. (2005) claim that SLs display high morphological complexity with respect to other young languages, like young creole languages. However, there is no agreement as to what constitutes a morpho-syntactic encoding in SLs and many phenomena previously likened to SpLs’ grammatical conventions are increasingly approached in indexical or depictive rather than descriptive, morphosyntactic, terms (see Puupponen, 2019). In the same vein, Schembri et al. (2018) argue that SLs exhibit little morphological complexity, partly because of their relatively young age. Despite the difficulty in determining the time depth of some SLs (de Vos and Nyst, 2018), understanding how SLs’ recent history impacts their morpho-syntactic structures will require both theoretical and methodological developments to identify criteria of complexity and the analysis of more SLs of different ages.

### On the Interconnectedness of the Frames

Each of the presented frames has been explained in isolation for the sake of clarity. However, causal processes at play in languaging occur simultaneously and are undoubtedly interrelated. To try and make sense of this multiplicity of biases on language, Enfield (2014, p. 17) proposes to ask: “*[H]ow might the outputs of processes foregrounded within any one of these explanatory frames serve as inputs for processes foregrounded within any of the others?*”. For instance, the link between ontogenetic factors and morphological complexity discussed above clearly involves many other causal frames. These include the microgenetic frame (e.g., the harder processing of a second language for late learners) and the enchronic frame (e.g., the contact situation which involves the intent to interact and, potentially, streamline language use). As an output, this ontogenetic difference leads to diachronic change (e.g., less complex uses spreading through a community).

Applying this reasoning to the comparison of SLs and SpLs, the sensory microgenetic difference has (only) one direct consequence that, in turn, impacts the languaging practices of signing communities: the easier availability of sound for hearing than for deaf individuals. Hence, one question that linguists interested in cross-modal differences should not miss relates to whether other factors may make signing communities different from other languaging communities. As seen above, these factors do exist. Another crucial question to ask relates to how this microgenetic factor serves as an input for processes foregrounded in other causal frames. For instance, what is its impact on the distribution of the three basic methods of communication found in all languaging communities (Clark, 1996; Ferrara and Hodge, 2018)? Ferrara and Hodge (2018, p. 12) propose that “*both signers and speakers signal through* [description,] *indication and depiction within the spatiotemporal context of their unfolding interactions, although the exact manifestations of these patterns diverge according to the availability of sound.*”

Bearing the prior considerations in mind, the next section looks at how enactment differences have been explained and rephrases these claims in light of Enfield’s terminology. While Enfield’s framework does not bring new explanations for cross-linguistic or cross-modal differences, it enriches language researchers’ conceptual toolbox by providing them with a new way of framing their explanations of these differences. It provides clear labels for sets of causes traditionally used in different sub-disciplines and research traditions that do not always engage with each other. This lack of interaction means that some accounts of cross-modal differences run the risk of neglecting some causal explanation or of ignoring how the causal frames they invoke interact with other causal processes. While not all frames listed by Enfield may be relevant to explain cross-modal differences, research on enactment can benefit from its innovativeness. The reframing of claims on enactment in light of these causal frames can highlight similarities and differences between different approaches to enactment and facilitate dialogue across researchers. In addition, it will be argued that research on enactment has neglected some causal frames that could help explain cross-modal differences.

### Accounting for Cross-Modal Differences in Enactment

Several approaches have been taken to interpret cross-modal differences in enactment. All resort to the well-known microgenetic modality difference. Noting the more frequent localisation of manual enactment predicates in DGS than German, Perniss and Özyürek (2015, p. 20) propose that “*the systematic use of space in the service of reference-tracking and discourse cohesion*” in SLs is due to the fact that “*the visual modality is used unimodally within a linguistic system.*” Signed language linguists have also proposed that patterns reported in studies on SL information packaging can be accounted for by noting that signers fully exploit the affordances of the visual-kinesic modality (e.g., Slonimska et al., 2020). How could this difference evolve in diachronic terms? Perniss et al. (2015, p. 7) propose that “*[i]n comparing sign and (co-speech) gesture from the perspective of conventionalization from gesture to sign, investigation of the degree of conventionalization can reveal new insights into lexicalization, linguisticization, and grammaticalization processes.*” Microgenetic-diachronic accounts postulate that SLs, because of their visual-kinesic modality, conventionalise or grammaticalise communicative actions also found in speakers’ gestures. For instance, Quer (2011) and Herrmann and Steinbach (2012, p. 223) offer an analysis of quotational enactment as resulting from a grammaticalisation process specific to SLs: “*[T]he development of role shift into a non-manual grammatical device systematically marking quotations seems to be a modality-specific characteristic of sign languages, which have the unique property of grammaticalizing manual and non-manual gestures.*” This unique property is attributed to the common modality of speakers’ gestures and SLs: “*Since gestures use the same articulatory channel that is also active in the production of signs, it is not uncommon for manual and non-manual gestures to become grammaticalized in sign languages*” (Herrmann and Steinbach, 2012, p. 222).

A second explanation for the differences observed between signers and speakers combines the microgenetic modality factor with enchrony. Considering all forms of enactment as depictive, the higher prevalence of this kind of depiction in SLs rather than SpLs could be due to the face-to-face nature of signed interactions and the cultural importance of storytelling in these communities (Marentette et al., 2004; Earis and Cormier, 2013). A similar point is made by Hodge and Ferrara (2014, p. 391): “*[A]s storytelling constitutes a conventional ‘script’ of expression for many Auslan signers across many communicative domains, we argue that enacted performance is ubiquitous within these signed language ecologies.*” Hence, from this perspective, signers and speakers’ differences are rooted in cultural norms about how acceptable and well-received enactment is as a semiotic strategy. A similar explanation has already been used to account for a related depictive phenomenon: the language used when reporting an utterance originally produced in a language not understood by one’s interlocutor. Evans (2012, p. 73) reports that one “*contribution of the speaker to the construction of ‘direct speech’ that we tend to take for granted* […] *is the translation of quotes into the language currently being used by the narrator.”* Evans shows that a narrator reports Dalabon speech in Dalabon in a Kriol narrative, despite the addressee’s lack of command of the language. Evans’ enchronic account of this difference is reminiscent of Ladd (2003)’s proposal about storytelling prestige for deaf communities: “*In many Aboriginal speech communities* […], *a good narrator will reproduce the language choice of the characters as accurately as possible, even where the hearer may not understand the quoted language*” (Evans, 2012, p. 73).

What do these causal models predict? If enactment undergoes grammaticalisation in SLs, one may expect paradigmatic differences between standard, depictive uses and their grammaticalised counterpart(s), like quotational enactment. Following grammaticalisation accounts, quotation should co-occur more often (or obligatorily) with enactment in SLs than in SpLs. In addition, signed quotational enactment should exhibit a fixed form-meaning pairing. This form should stand in contrast with its non-quotational counterpart in SLs or any form (quotational or otherwise) of enactment in SpLs. For instance, one could expect a constrained set of articulators, perhaps systematically articulated in specific manners (see Herrmann and Steinbach, 2012; Herrmann and Pendzich, 2018; Quer, 2019). Indeed, Steinbach (2021, p. 356) proposes that

*[P]rototypical cases of AtRS* [attitude role shift, i.e., quotational enactment] and *AcRS* [action role shift, i.e., non-quotational enactment] *have different functional and formal properties: while AtRS is used to report utterances, thoughts, or attitudes and thus includes mainly linguistic material (typically sentences denoting propositions), AcRS is used to report actions and includes mainly gestural demonstrations. Furthermore, both kinds of role shift differ in their non-manual marking.*

In contrast, by the second account, enactment by signers and speakers alike is an act of selective depiction, that is, a form of improvised semiotics (Hodge and Ferrara, 2014). Cross-modal differences can then rather be explained by social-interactional, cultural differences. Variation exists in members of a community’s observance of social norms. Enfield (2014, p. 33) refers to Gladwell’s (2000) considerations about personality traits:

*[D]ifferent personality types contribute to the diffusion of innovation in complementary ways. Connectors have a high number of weak social connections, in a range of social spheres. Mavens are actively interested in the market, and want to share their knowledge and opinions. Salesmen are the charismatic, persuasive ones who model innovations and effectively sell them. Innovators are the risk-takers who try things before anyone else does. They are followed by early adopters, the early majority, the more conservative late majority, and finally, the laggards.*

If enactment is one such social norm, one could expect intra- and interindividual variation in its use. No constraints of obligatoriness would be found for either the use of specific articulators or specific manners of articulation. Rather, these characteristics would be driven by context-dependent factors like the nature of the target referent, referential salience, stylistic choices or common ground with one’s addressee. Focusing on reported speech, Genetti (2011, p. 73)’s comment on the enacting use of voice adopts a similar view: “*[O]ne needs to consider inter-speaker variation in style. Speakers vary in their interest and proficiency in storytelling and in the degree to which they use a performative style.*” As phrased by Kimmelman and Khristoforova (2018, p. 101), “[*t*]*he optionality of non-manual marking can be explained by the variation in how precise and how expressive the signer decided to be when quoting someone.*” Taking a step further in microgenetic-enchronic predictions, if speakers and signers differ because of social-interactional norms of depiction, one could predict that observed differences hold for both quotational and non-quotational uses of enactment. For instance, taking for granted that utterance reporting is a form of enactment and that enactment is more frequent in some SLs with respect to their ambient SpLs, signers might use utterance reporting more frequently than speakers for the same SL-SpL pairs.

The different causal accounts presented above are not mutually exclusive. It may well be that signers and speakers differ both in their cultural appreciation for enactment and the extent to which this strategy has become conventionalised to express certain meanings in different languages, spoken or signed. Researchers arguing for a conventionalisation phenomenon in SLs acknowledge that teasing apart depictive enactment from its potential conventionalised offshoots is no easy task. Quer (2011, p. 287), for instance, says: “*As a consequence of the language modality, both regularly coexist, either simultaneously or consecutively. Although sometimes the limiting line between the two sorts of elements is hard to draw* […], *I would like to defend that it exists.*” In the rest of this paper, I expand on the idea that, to move the debate forwards, the predictions derived from these claims can and should be tested. Thanks to the creation of comparable SL and SpL corpora, language researchers can now avail themselves of better data to make robust claims on the aspects in which signers and speakers differ and pinpoint those features of enactment which are best explained enchronically and those which could result from a conventionalisation phenomenon. In the next sections, this paper argues in favour of this recent methodological contribution to complement current approaches used to study enactment.

## Documenting Enactment Cross-Modally

Drawing on Stefanowitsch (2020)’s discussion of the criteria of authenticity, diversity and size as well as several remarks on the suitability of corpus linguistics for the study of SLs, the following section addresses shortcomings in the literature and advocates for a corpus-based comparative approach. First, one limitation of some works lies in the absence of data, or reports of the used data, on which the claims are made. Indeed, some statements on the use of enactment seem to draw on researchers’ intuitions about speakers’ use of depiction. To really understand cross-modal differences, comparable data is needed. Hodge et al. (2019) introduce the Auslan and Australian English archive and corpus, the first directly comparable set of corpora of a SL and its ambient SpL. Thanks to similar sampling frames, the communicative practices of both languaging communities can be directly compared using authentic and diverse language use. The constitution of such multilingual corpora is timely: the corpus-based approach to enactment, as shown in this review, is well on its way and the use of enactment as a comparative concept allows for its operationalisation in corpus studies with well-defined, reproducible, annotation procedures. Guidelines for the study of enactment drawn from Cormier et al. (2015), for instance, have been applied in different studies (e.g., Slonimska et al., 2021; Vandenitte, 2021; Puupponen et al., 2022).

How frequently do users of different languages use enactment? How often do they use specific articulators such as their hands, lower half of the body, non-manuals or voice? What are the manners of articulation of these articulators and how often are they used? What is the impact of modality and how does it interact with the physical properties of the intended referent or action (e.g., quotational vs. non-quotational enactment), culture, genre, register, discourse salience of the referential target or individual style? More specific examples of questions that lie ahead of enactment researchers and could be answered by such corpora, include: What kinds of referents do speakers and signers enact? Could it be that the availability of voice for SpL enactment leads to a different distribution of articulator use with respect to SLs? As a considerable part of descriptive meaning-making relies on different channels for SLs and SpLs, does that have an impact on the articulators they use for enactment? To what extent does enactment provide core meaning contributions cross-modally? Do speakers also use enactment to make their communication more efficient or is the strategy mostly a narrative and/or evaluative one in SpLs? Are role-shift practices the same in SLs and SpLs? Are they specific to quotational contexts or are they equally found whenever an interaction between two referents is enacted, regardless of whether that interaction involves a languaging event? Studies aiming at answering these questions should strive towards meeting several conditions that are detailed in the next sections.

### Sampling Enactment in More but Mostly Diverse Languages

A better understanding of language diversity and its causes can be reached by comparing several language pairs and ensuring diversity in the profiles of the communities who sign or speak these languages. When it comes to cross-modal comparisons of enactment, it may well be that the few SpLs studied by linguists so far, such as American English, are not as gesture-rich as others, e.g., Italian (Iverson et al., 2008). This call for research on diverse languaging communities is in line with Zeshan and Palfreyman’s (2020, p. 530) agenda for the emerging field of cross-modal typology, “*typological research in linguistics that takes into account the differences and the commonalities that exist both between languages and across the two modalities of signed and spoken language.*” It is indeed well-known that certain communities and languages have been studied more than others. Henrich et al. (2010, p. 1) have shown that researchers in the fields of behavioural sciences have focused on WEIRD communities: “*Western, Educated, Industrialized, Rich and Democratic.*” Majid and Levinson (2010) argue that linguistics is similarly biased: research in this field has taken for granted that claims that could be made for the languages of WEIRD communities, such as English, could equally be applied to other, non-WEIRD languages. The study of enactment too seems to have been mostly confined to a subset of communities. SpLs in which enactment has been studied include Arabic, English, German, Greek, Japanese, Korean, Spanish, i.e., languages spoken by large communities (Tannen, 1986; Cameron, 1998; Park, 2009; Earis and Cormier, 2013; Thompson and Suzuki, 2014; Quinto-Pozos and Parrill, 2015; Stec et al., 2016; Bressem et al., 2018; Soulaimani, 2018).

Similarly, most research on SLs is restricted to those signed in Europe and North America, particularly languages of signing macro-communities (Fenlon and Wilkinson, 2015, p. 7). These languages “*are transmitted primarily through peers at schools or are learned later in life. They are minority languages surrounded by majority-SpLs, consist of both deaf and hearing signers, and are young languages*” (Fenlon and Wilkinson, 2015, p. 9). Even though macro-community SLs have been the focus of most works in signed language linguistics, they are but one part of the signing communities around the globe. Other communities also use SLs as (one of) their primary languages: these languages are those signed by micro-communities, “*characterized as small labour-intensive economy-based communities, with a much higher incidence of deafness than that seen in developed countries and urban communities*” (Fenlon and Wilkinson, 2015, p. 9). As a consequence, in these communities, there is a “*high number of deaf signers and hearing signers living in close proximity [and] deaf children are much more likely to acquire a signed language from signing parents or from other extended family members and neighbours who can sign*” (Fenlon and Wilkinson, 2015, p. 10). Most SLs in which enactment has been studied are macro-community SLs such as ASL, Auslan, BSL, DGS, DTS (Danish Sign Language), FinSL, Libras (Brazilian Sign Language), LSC (Catalan Sign Language), LSF (French Sign Language), LSFB, LSQ (Quebec Sign language), and SASL (South African Sign Language) (Cuxac, 2000; Aarons and Morgan, 2003; Janzen, 2004; Meurant, 2008; McCleary and Viotti, 2010; Quer, 2011; Herrmann and Steinbach, 2012; Earis and Cormier, 2013; Hodge and Ferrara, 2014; Engberg-Pedersen, 2015; Quinto-Pozos and Parrill, 2015; Parisot and Saunders, 2019; Jantunen et al., 2020).

Progress towards capturing enactment diversity has recently been made by two breakthroughs: the inclusion of less WEIRD communities and the introduction of cross-linguistic and cross-modal studies. The highly diverse profiles of both speaking and signing communities may provide keys to grasp the causes underlying semiotic diversity because they feature different combinations of several factors shaping language use and structure. As Zeshan and Palfreyman (2020, p. 533) suggest, ‘*it may be argued that sign languages are not different from spoken languages per se, but pattern with particular sub-types of spoken languages*’. For instance, studying micro-community SLs, for which transmission patterns are closer to SpLs where all children acquire at least one directly accessible language from birth, may allow to control for ontogenetic causes in a comparison. Similarly, including SpLs of communities with a strong oral culture helps unravel the impact of enchronic factors on enactment. A striking example that, to the best of my knowledge, has not yet made its way to the signed language linguistics literature, is that of Chantyal, a language from the Tibeto-Burman family found in Nepal. The descriptions provided by Noonan (2006, p. 1) of Chantyal speakers’ use of “*direct speech as a rhetorical speech*” are reminiscent of frequent claims on SL enactment. One is that tokens of direct reported speech are frequent in Chantyal (p. 24). Another is that this strategy is crucial in Chantyal discourse. Noonan refers to a potential rephrasing of Chantyal speech that would exclude direct reporting: “*While such a discourse would be fully grammatical, it would be* […] *decidedly unidiomatic. Part of being a fluent speaker of Chantyal involves knowing how and when to use quotatives. Quotatives constitute part of the ‘flavor’ or ‘style’ of the language*” (p. 27). Noonan analyses this frequent use of reported speech in enchronic terms: “*The effects that rhetorical styles produce are ultimately social and interactional in origin and not specifically grammatical*” (p. 30). Though empirical comparisons are required, one could be tempted to say that, as far as (quotational) enactment is concerned, Chantyal is a good candidate, to reuse Zeshan and Palfreyman’s phrasing, for those sub-types of SpLs with which SLs pattern. Other examples of less WEIRD languages in which enactment has been studied include Murrinh-Patha (Blythe, 2009), ISN (Nicaraguan Sign Language) (Kocab et al., 2015), as well as ABSL (Al-Sayyid Bedouin Sign Language) and KQSL (Kufr Qassem Sign Language) (Stamp and Sandler, 2021).

Among these, comparative research between SLs with different sociolinguistic profiles have aimed at better understanding enactment diversity. For instance, Pyers and Senghas (2007) compare ISN to ASL, two macro-community SLs which differ in age. ASL is an older language (about 200 years old) whereas ISN is still referred to as “emerging.” Pyers and Senghas (2007) note that ASL and ISN enactment are alike in the frequent break in gaze address but differ in the way the upper body is used to enact referents: whereas ASL signers resort to a lateral lean of their shoulders, ISN signers would rotate their torsos. Neither methodological explanations to distinguish between the two movements nor quantitative support for this claim are however provided. Kocab et al. (2015) compared the first and the second cohorts of ISN signers to investigate whether there would be a different use of torso movements across cohorts due to the grammaticalisation of enactment. They note that, “*[c]ompared to the first-cohort signers, second-cohort signers used significantly more [*…*] body shifts*” (Kocab et al., 2015, p. 9). However, no account of how depictive and grammaticalised torso movements were distinguished is provided either. In a more recent study, Stamp and Sandler (2021) compare two micro-community SLs with different network densities, ABSL and KQSL, with a macro-community SL, ISL (Israeli Sign Language). They note that what they consider to be depictive enactment is found across all three communities. They do however differ in the use of conventionalised strategies of enactment, which they classify as “*complex abstract forms*” (Stamp and Sandler, 2021, p. 11), where body positions indicate rather than depict referents. They find that ISL signers use more indicating body shift than ABSL and KQSL signers, who prefer depictive enactment. This difference may be explained by the fact that ISL is a macro- rather than a micro-community SL. Because ISL is a less close-knit community than ABSL and KQSL, Stamp and Sandler propose that it could be under more pressure for conventionalisation. Since these works have relied on the description of short vignettes by participants, it would be interesting to investigate how the ISL, ABSL, and KQSL communities use enactment in spontaneous interactions.

As shown earlier, most cross-modal comparisons of the phenomenon have been limited to macro-community SLs and their ambient SpLs, such as BSL-British English, DGS-German, and ASL-American English (e.g., Earis and Cormier, 2013; Perniss and Özyürek, 2015; Quinto-Pozos and Parrill, 2015). One recent exception is Hodge, Barth and Reed’s (under review) comparison between Auslan and Matukar Panau, an Oceanic SpL of Papua New Guinea. Auslan and Matukar Panau are comparable in several respects. Both are face-to-face languages and their communities form tight social clusters. Both languages are also surrounded by another, majority language (Australian English and Tok Pisin). The comparison shows that Auslan signers use enactment about three times more often than Matukar Panau speakers do. Another difference lies in that Matukar Panau speakers mostly enacted referents as speaking or thinking in the study whereas Auslan signers more frequently enacted referents as doing or thinking, and only sometimes dialoguing. In addition, the forms of enactment are partly different in the two communities. Auslan signers used visible forms whereas Matukar Panau speakers preferred to enact referents using their voice. Even when Matukar Panau speakers did use visible bodily forms of enactment, differences arise between the two language groups: they recruited different sets of articulators and Auslan signers tended to use more articulators on average (e.g., using both their head and face rather than using only one of these articulators). In addition to patterns specific to Auslan or Matukar Panau, Hodge et al. also stress interesting variation within each language group. By comparing a SL to a SpL that is not its ambient SpL as well as by addressing individual variation, this study adds to the understanding of enactment in a way not achieved in prior comparisons. Further work is crucial to include signing and speaking communities which differ from each other in ontogenetic, enchronic and diachronic terms. Now that a case has been made for the inclusion of a more diverse sample of languages, specific methodological issues found in the literature are addressed.

### Towards Better (Enactment) Data

#### Size

Most comparative studies of enactment have focused on small-scale, though fine-grained, analyses. For instance, Rayman’s (1999) study is based on the use of enactment by 5 ASL signers and 5 English speakers. Both Marentette et al.’s (2004) and Perniss and Özyürek’s (2015) comparisons studied enactment as produced by 8 ASL/DGS signers and 8 English/German speakers. In Earis and Cormier (2013), the phenomenon is compared across 2 BSL signers and 2 English speakers. In Quinto-Pozos and Parrill’s (2015) study, the number of participants is the highest (23 English speakers and 10 ASL signers). Analysing large samples of data is crucial to study the frequency of use of enactment, the articulators it recruits and their manners of articulation. Large-scale studies may provide solid accounts of variation patterns and their potential causes by distinguishing individual from community patterns (Barth et al., 2022). This is crucial for macro-community SLs because of the unique sociolinguistic ecology of these signing communities and their subsequent highly variable patterns of language use. As put by Fenlon et al. (2015, p. 158),

*[T]he variability owes much to the fact that SLs exist in unique sociolinguistic circumstances: they are young, minority languages, with few native signers and with an interrupted pattern of intergenerational transmission. As a consequence, it is often difficult even for native signers to be certain about what is and is not an acceptable construction in their language. [P]rocessing* […] *large amounts of annotated texts can reveal patterns of language use and structure not available to everyday user intuitions, or even to expert detailed analysis.’*

A large corpus is more likely to contain many tokens of enactment and hence show potential patterns of variation across different uses of the phenomenon. Large-scale analyses are thus an ideal way to distinguish idiosyncratic variation from patterns common to larger groups in the community or to the whole community.

#### Authenticity

Next, taking for granted that authentic data is key to reliable insights on natural language use (Kusters and Hou, 2020; Stefanowitsch, 2020), one can wonder how the experimental setting of some studies impacts enactment and its authenticity. As noted by Stec et al. (2016), authenticity in prior comparisons of enactment could be improved by ensuring dialogic, spontaneous language use by participants with no specific storytelling experience. Participants in Earis and Cormier’s (2013) study received a summary of the story to be told in advance and were given time to prepare for their storytelling task. Highly controlled narrative retelling tasks using elicitation materials like cartoons or other visual stimuli may also undermine authenticity with respect to less controlled settings, such as one where topic choice is left to participants. Furthermore, taking for granted that interaction is the natural locus of language use (Clark, 1996), monologic language use, like that produced by participants in Earis and Cormier’s (2013), could impact the use of enactment. Indeed, Bavelas et al. (2014) have shown that monologues feature significantly less depiction than dialogues. In a similar vein, having a researcher be the participants’ interlocutor, such as in Rayman’s (1999) study, might lead to less authenticity.

#### Representativeness/Diversity

Lastly, another shortcoming in the literature is the lack of representativeness. To make generalisations on communicative phenomena, it is important for the sample to be as representative of language use in its totality as possible. However, as Stefanowitsch (2020) shows, representativity is rarely possible and the best next option is that of diversity. A similar point is raised by Fenlon et al. (2015, pp. 160-161) for SL corpora in particular:

*[P]articipants are selected as part of a quota sample, according to a set of demographic variables (e.g., gender, age, region, ethnicity, socioeconomic class, and age of SL acquisition) that are considered relevant to deaf communities. Although the resulting data set may or may not be representative of the wider deaf community* […], *recruiting participants via a quota sample with these demographic variables does take us some way towards capturing the full range of variability in the deaf community.*

Accounts of language use at the community level are thus best achieved by putting the emphasis on the diversity of the data sample. This point bears particular relevance when it comes to deaf signing communities because of the heterogeneous profiles of their members. As enactment has been described as harder to command for late hearing learners of a SL (Gulamani et al., 2020), it is relevant to ask what impact one’s language acquisition profile has on one’s use of enactment. Future studies could compare how native signers differ from near native and late signers, for instance. Fenlon et al. (2015, p. 164) emphasise that text type constitutes another layer of diversity in the data sample: “*While there are some differences between projects in the type of data collected, there is a clear consensus among projects that different genre types should be sampled in order to maximize representativeness.*”

Fenlon et al. (2015) show that narrative tasks are well represented in SL corpora. Coming back to the study of enactment, more diversity could prove fruitful to see how the phenomenon varies across different discourse genres. Indeed, most works have largely concentrated on narrative data (Slonimska et al., 2021). Participants are often asked to perform a narrative retelling task and, in two cases (Rayman, 1999; Earis and Cormier, 2013), at least some participants or all were known to be highly skilled storytellers. The inclusion of narration is interesting as it is known to be a prevalent discourse genre, potentially more so for signing communities than for speaking ones (Ladd, 2003; Hodge and Ferrara, 2014). However, by focusing almost exclusively on narration, research on enactment fails to meet the diversity criterion as it is left unknown how the use of enactment might compare across different genres, like argumentation or description (see Puupponen et al., 2022 for a recent exception). Because of issues related to intuition-based claims as well as improvable sizes, degrees of authenticity and diversity of the investigated datasets, further comparative research aiming at avoiding these pitfalls is warranted. Ensuring corpus diversity in language users’ profiles and in their linguistic activities should help provide a clearer account of intra- and inter-individual variation and better understand why and how they use enactment in specific contexts.

## Discussion

Several questions remain open as to the diversity of ways signers and speakers of different communities use enactment. While enactment is more frequent in SLs than SpLs, the amount of idiosyncrasy and conventionality in enactment forms remains unclear: Are some articulators and manners of articulation systematically recruited in community-specific ways and for particular communicative functions? For instance, the distinction between quotational and non-quotational uses of enactment in SLs has yet to be empirically supported. Which are those articulators and articulatory behaviours specific to either SLs or SpLs?

In this paper, I have highlighted conceptual and methodological avenues for cross-linguistic and cross-modal comparative research on enactment. Explaining cross-modal differences should be done by recognising that the modality used by the community is not the only factor at play. In addition, this factor should always be considered in pair with adjacent causal inputs and causal processes for which it serves as an input. What impact do other factors such as time depth of a language (diachrony), community-specific social-interactional norms (enchrony), age of language acquisition (ontogeny), and their interactions, have on the use of enactment?

Main accounts of enactment differences between SLs and SpLs have been reformulated in Enfield’s terms. The diachronic grammaticalisation account proposes that a semiotic shift away from depiction occurs, whereby some uses of enactment, like quotational uses, can be described as fixed form-meaning pairs in SLs. The enchronic account views both uses of enactment as equally depictive, whereby utterance reporting constitutes one sub-kind of enactment where the reported utterance is depicted. The predictions of both these approaches have been fleshed out for further comparative research. On the one hand, the grammaticalisation account predicts that some formal aspect of enactment should be conventionalised for a specific function (e.g., indicating a reported utterer) across discourse genres in a community-specific way. On the other hand, the enchronic account predicts intra- and inter-individual stylistic variation as well as an impact of local factors like discourse genre, intended referent or discourse salience. Following the enchronic approach, quotational and non-quotational uses of enactment should look similar. In addition, if utterance reporting is a sub-kind of enactment, its use could be more frequent in those communities where enactment is generally more appreciated in interaction.

Finally, I have also argued that better enactment data is needed to empirically test these hypotheses. Therefore, comparisons of large datasets of spontaneous, interactional and diverse language use would be fruitful for the study of enactment. These comparisons should address multiple languages of diverse communities featuring different combinations of the microgenetic, ontogenetic, enchronic and diachronic causal factors. Only then will it be possible to spell out data-based accounts of how and why enactment differs across communities.

## Author Contributions

The author confirms being the sole contributor of this work and has approved it for publication.

## Conflict of Interest

The author declares that the research was conducted in the absence of any commercial or financial relationships that could be construed as a potential conflict of interest.

## Publisher’s Note

All claims expressed in this article are solely those of the authors and do not necessarily represent those of their affiliated organizations, or those of the publisher, the editors and the reviewers. Any product that may be evaluated in this article, or claim that may be made by its manufacturer, is not guaranteed or endorsed by the publisher.

## Footnotes

1 “Languaging” is here defined as the use of a multimodal and composite set of signals involving speech, sign, and speakers’ visible bodily actions. Languaging is thus a process that users of both signed and spoken languages engage in.

2 As conventional in signed language linguistics, ID-glosses in SMALL CAPS are used for signers’ manual communicative actions. These glosses are arbitrarily based on French words, here translated in English, which are not necessarily translations of the LSFB signs. “CA” stands for “constructed action,” another term for enactment. “LH” and “RH” respectively stand for “left hand” and “right hand”.

3 “Utterance reporting” and “quotation” will be preferred to “speech reports” and the like in the present paper because they are less modality-bound. In particular, these terms are used here to refer to what is commonly labelled as “direct speech reports” (notwithstanding that this excludes SLs) where the report features the reported utterer’s perspective (Coulmas, 1986). This focus on direct reporting is motivated by the fact that enactment also brings the enacted referent’s internal perspective to the fore.

4 For the sake of clarity, Herrmann and Steinbach’s (2012) use of “signer” could be substituted by “utterer.” Indeed, signers also enact/report SpL utterances.
